# Global 1 km × 1 km gridded revised real gross domestic product and electricity consumption during 1992–2019 based on calibrated nighttime light data

**DOI:** 10.1038/s41597-022-01322-5

**Published:** 2022-05-12

**Authors:** Jiandong Chen, Ming Gao, Shulei Cheng, Wenxuan Hou, Malin Song, Xin Liu, Yu Liu

**Affiliations:** 1 School of Public Administration, Southwestern University of Finance and Economics, Chengdu, China; 2 School of Finance, Shanghai Lixin University of Accounting and Finance, Shanghai, China; 3 University of Edinburgh Business School, University of Edinburgh, 29 Buccleuch Place, Edinburgh, Scotland; 4 School of Statistics and Applied Mathematics, Anhui University of Finance and Economics, Bengbu, China; 5 Curtin University Sustainability Policy (CUSP) Institute, School of Design and the Built Environment, Curtin University, Perth, Australia; 6 Institutes of Science and Development, Chinese Academy of Sciences, Beijing 100190, China; 7 School of Public Policy and Management, University of Chinese Academy of Sciences, Beijing 100049, China

**Keywords:** Developing world, Economics

## Abstract

As fundamental data, gross domestic product (GDP) and electricity consumption can be used to effectively evaluate economic status and living standards of residents. Some scholars have estimated gridded GDP and electricity consumption. However, such gridded data have shortcomings, including overestimating real GDP growth, ignoring the heterogeneity of the spatiotemporal dynamics of the grid, and limited time-span. Simultaneously, the Defense Meteorological Satellite Program’s Operational Linescan System (DMSP/OLS) and National Polar-orbiting Partnership’s Visible Infrared Imaging Radiometer (NPP/VIIRS) nighttime light data, adopted in these studies as a proxy tool, still facing shortcomings, such as imperfect matching results, discontinuity in temporal and spatial changes. In this study, we employed a series of methods, such as a particle swarm optimization-back propagation (PSO-BP) algorithm, to unify the scales of DMSP/OLS and NPP/VIIRS images and obtain continuous 1 km × 1 km gridded nighttime light data during 1992–2019. Subsequently, from a revised real growth perspective, we employed a top-down method to calculate global 1 km × 1 km gridded revised real GDP and electricity consumption during 1992–2019 based on our calibrated nighttime light data.

## Abstract (summary)

**Table:** 

Measurement(s)	GDP • electricty consumption
Technology Type(s)	machine learning

## Background & Summary

Economic activities are essential for human survival and development^1^. Facilitated by social organization and order, humans use labor and other production resources to exchange goods and services to create, transform, and achieve economic value. The economic output in a country or region is its accumulation of created economic values within a certain period; of which, Gross Domestic Product (GDP), is the most prevalent indicator globally. In addition, electricity consumption is adopted as a supplementary indicator that indirectly reflects the economic status and living quality of residents, because it is indispensable for industrialization^1,2^ and in daily lives of modern residents. In particular, the extensive consumption of electricity across different industries and sectors, makes it a substantial contributor to economic growth^3,4^. Numerous studies have reported that the availability of electricity for consumption enhances impoverished populations’ livelihoods, with consumption patterns reflecting quality of life and lifestyle^5,6^. Therefore, numerous scholars have heeded to the accounts of GDP, electricity consumption and their spatiotemporal change^7–9^. For example, Henderson *et al*.^10^ used the Defense Meteorological Satellite Program’s Operational Linescan System (DMSP/OLS) nighttime light data to revise real-world countries’ real GDP growth. Guerrero *et al*.^11^ proposed an improved method to revise a single country’s real GDP growth-based DMSP/OLS data. Wang *et al*.^12^ analyzed the driving forces behind electricity consumption growth in China’s industrial sector. However, most studies have been based on specific administrative region, such that detailed spatiotemporal changes in GDP and electricity consumption on a micro-level scale (e.g., small towns, villages and business clusters) are not easily accessible^13^.

Thus, a few studies have estimated gridded GDP and electricity consumption with specific resolutions based on several proxy tools. Gridded population and nighttime light data are the most popular proxy tools, and have been adopted extensively because of their strong correlation with economic output and electricity use. For example, Kummu *et al*.^14^ combined sub-national average GDP per capita with gridded population data to estimate gridded GDP data during 1990–2015. Similarly, based on the regional ratio of GDP to nighttime light data, Zhao *et al*.^15^ and Wang *et al*.^16^ calculated China’s 1 km × 1 km gridded GDP during 2000–2015. With the regard to gridded electricity consumption, Shi *et al*.^17^ used DMSP/OLS nighttime light data to estimate gridded electricity consumption at 1-km resolution during 1992–2013. Similarly, Chen *et al*.^18^ employed a classification regression method to calculate China’s electricity consumption in 2015 based on a combination of NPP/VIIRS data and land use and cover change (LUCC) data for 2015.

Considering existing literature, several limitations of estimated gridded GDP and electricity consumption datasets have been noted. (1) In many studies, gridded GDP data are estimated solely based on official GDP statistics; however, the published GDP growth of some countries, especially developing countries (e.g., China^19^ and some African countries^20^), may have errors due to poor statistical methods or intentional manipulation^10,21^. Although nighttime light data, as a globally comparable, objective, and highly economical instrumental variable, has been widely used to correct economic growth data^10,11^, it has not been applied in existing research on gridded GDP measurements. Actually, most studies on gridded GDP estimations directly used gridded nighttime light intensity as the distribution weight for allocating a country’s official GDP and calculating the raster data^14–16^. Hence, the grid GDP growth rate may be inaccurate in such studies. (2) Most gridded GDP data are based on the hypothesis that the ratio of GDP to nighttime light data are identical in the same countries, ignoring the grid heterogeneity over space. (3) Moreover, due to the gap between DMSP/OLS and NPP/VIIRS products, long time spans of estimated gridded GDP based on the nighttime light data are limited. (4) Furthermore, the time spans of existing datasets are short and outdated, and do not match those of other updated data. Similarly, for gridded electricity consumption data: (1) due to the limited availability of electricity consumption and nighttime light data, the long-span data was only obtained up to 2013; and (2) based on the hypothesis that the ratio of electricity consumption to nighttime light data are identical in regions, the models adopted in the calculations of electricity were simple, thereby failing to capture the grid heterogeneity over space.

Although nighttime light data as a single indicator may ignore factors such as value added or reduced by forestry or desertification, it is still an effective proxy for calibrating economic growth^22^. The influence of neglected factors on total economic output is limited, and night lighting, as satellite data, has advantages that other indicators (e.g., gridded population data) cannot surpass, such as objectivity, wide range, and high correlation with economic indicators^23^. DMSP/OLS (1992–2013)^24^ and NPP/VIIRS (2012–)^25^ images are widely used nighttime light data, owing to their long timespans, wide space coverage, and ease of obtaining and updating. However, there are apparent gaps between the two sets of nighttime light data, which hinders the wider application of long-term, continuous nighttime light data. Specifically, the gap between the pixel-level values of DMSP/OLS and NPP/VIIRS images in 2013 could primarily be attributed to the inconsistency at the time of observation, different sensors and cloud cover, which caused ‘high–low’ or ‘low–high’ problems in the pixel values of the two images^26^ (i.e., the pixels in the NPP/VIIRS image have a high [low] DN value in 2013, whereas the pixels in the same place have a low [high] DN value in the DMSP/OLS image). Several studies have attempted to unify the two sets of satellite data at the pixel level^27,28^. However, the matching process has proved difficult, and the results show low fitting and discontinuity in temporal and spatial changes, leaving room for improvements.

Therefore, this study proposed an improved approach to unify the scale of DMSP/OLS and NPP/VIIRS images, and obtained continuous and stable calibrated nighttime light data during 1992–2019, that are better fitting than those in existing literature. Subsequently, from a real growth rate perspective, we estimated the global 1 km × 1 km gridded revised real GDP and electricity consumption based on the top-down method optimized using the Particle Swarm Optimization-Back Propagation (PSO-BP) algorithm. The datasets provided in our study enrich basic data for research on economics, management, and other issues. Simultaneously, considering our gridded GDP growth was revised based on nighttime light data, it is more objective and comparable, and can be applied in research at the micro-level around the world (especially in some countries with poor statistical quality).

## Methods

### Study areas and Data preprocessing

Given that the estimations were based on the top-down approach, the study areas depended on the countries that provided the available data. The GDP data includes 175 countries (or regions), and the electricity consumption data includes 134 countries (or regions). As such, the research scope covers over 70% of the global land area, and over 90% of the GDP and electricity consumption.

Two sets of nighttime light data were used in this study: Defense Meteorological Satellite Program/Operational Linescan System (DMSP/OLS) and National Polar-Orbiting Partnership’s Visible Infrared Imaging Radiometer Suite (NPP/VIIRS) images. Considering the versions of nighttime light data, we selected annual stable DMSP/OLS images after removing noise and monthly NPP/VIIRS images without cloud cover, because they have better fitting effects with economic output and other socioeconomic factors. The DMSP/OLS resolution is approximately 1000 m and it comprises six different DMSP satellites F10 (1992–1994), F12 (1994–1999), F14 (1997–2003), F15 (2000–2007), F16 (2004–2009), and F18 (2010–2013). The geographic coordinate reference system of the DMSP/OLS image is the WGS-84 coordinate system, the acquisition width is 3000 km, and the spatial resolution is 30 arc seconds (approximately 1 km near the equator and 0.8 km at 40° north latitude). The coverage of the image is from −180° to 180° in longitude and from −65° to 75° in latitude (covering all areas of the world where human activities exist). The spatial resolution of the NPP/VIIRS image data was higher than that of the DMSP/OLS image, which was 413 m. Simultaneously, unlike DMSP/OLS images that only provide relative radiation values in the range of 0–63, NPP/VIIRS images provide absolute radiation values in the unit of Watts/cm^2^/sr. Considering that there are several problems in satellite images, such as saturation, discontinuities, and white noise, these datasets needed to be pre-processed before they could be used further.

With regard to DMSP/OLS images, we projected the images as a Mollweide projection and resampled them at a spatial resolution of 1 km. Next, based on the invariant region method, we adopted the form of a power function to reduce saturation. In light of the power function parameters provided by Shi *et al*.^17^, the images were calibrated. Given that the two sensors both provided images in specific years (e.g., F10-1994 and F12-1994), we averaged them to obtain individual images for each year. As for the discontinuities, annual continuous processing was adopted based on the assumption that the stable DN value of a pixel on the light image in the following year should not be less than the stable DN value of the pixel in the previous year^29^.

For the NPP/VIIRS images, we adopted 0.3 Watts/cm2/sr as the threshold to remove the noise, which is consistent with previous studies^26,30^. To avoid the influence of stray light pollution in summer, monthly images from June to August were removed. Next, based on the average monthly data, we estimated the annual NPP/VIIRS images from 2014 to 2019. As for the discontinuities, we also adopted the same annual continuous processing with DMSP/OLS images. Finally, to better match the DMSP/OLS image, we resampled the NPP/VIIRS image from a resolution of 0.5 km × 0.5 km to that of 1 km × 1 km.

### Matching of the two sets of nighttime light data

The gap between DMSP/OLS and NPP/VIIRS images is mainly driven by different sensors, spread functions, and spatial and temporal inconsistence^21^. Considering that the relationship between the two data sets is like a “Black Box,” Chen *et al*.^31^ used an artificial neural network (ANN) to explore the potential functions on the two data sets, and the matching results proved successful. Based on their study, we also employed a particle swarm optimization-back propagation (PSO-BP) algorithm to unify the scale of DMSP/OLS and NPP/VIIRS images. The initial parameters of the PSO-BP algorithm (i.e., C1 and C2 values were both set to 2.0, and the structure of the model included one hidden layer with five nodes; the maximum iteration number and population size were set to 50 and 20, respectively) were set following Chen *et al*.^31^.

Moreover, because our target is pixel-level matching, errors of hundreds of millions of pixels make the matching effect very poor, even after using machine learning. The difficulty is mainly driven by the ‘high–low’ or ‘low–high’ problems in the pixel DN values of the two images^26^ (i.e., the pixels in the NPP/VIIRS image have a high (low) DN value in 2013, whereas the pixels in the same place have a low (high) DN value in the DMSP/OLS image). Therefore, we proposed the principle of ‘high to high’ and ‘low to low’ for the matching job.

**Fig. 1:**
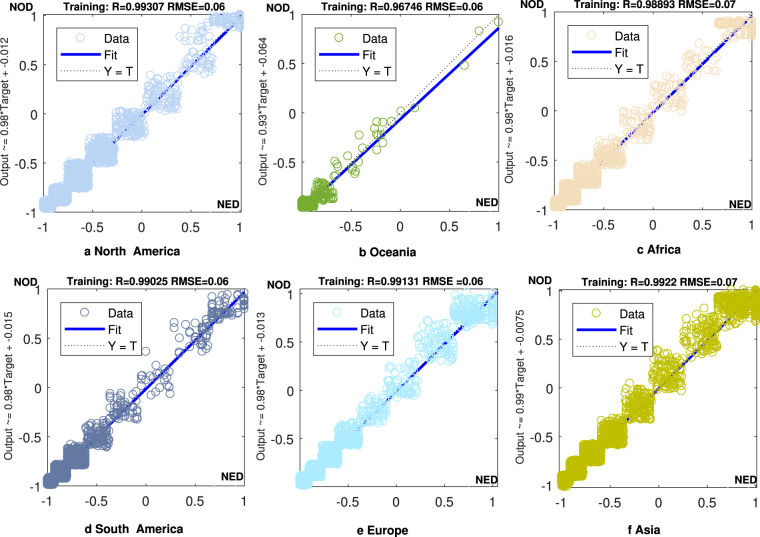
Different training results for the pixel normalized values on the six continents. (**a**–**f)**. Training results for the pixel normalized values on (**a**) North America, (**b**) Oceania, (**c**) Africa, (**d**) South America, (**e**) Europe, and (**f**) Asia.

Thus, we divided the DMSP/OLS and NPP/VIIRS images into nine categories based on the natural interval method. By matching similar attributes in the two images, we extracted and obtained sampling points that met the principles of ‘high to high’ and ‘low to low’ in the analysis. Subsequently, in line with Chen *et al*.^31^ and Li *et al*.^28^, we used the logarithmic form of the pixel DN values in the NPP/VIIRS image in 2013, and their latitude and longitude as input factors. The DN values of the pixels in the DMSP/OLS image in 2013 were selected as the output factors. In addition, according to general practice in machine learning^28,31^, the input and output factors were normalized to avoid the influence of indicators’ units. Considering the continental heterogeneity, we estimated six continental parameters of the PSO-BP neural network (e.g., North America, South America, Oceania, Africa, Asia, and Europe). Antarctica was not considered in this study because the scope of sensors that provided DMSP/OLS and NPP/VIIRS images did not include Antarctica. The matching results based on the training sets (60% of the total samples) are presented in Fig. 1. In particular, the NED in the x-axis represents normalized DN values converted from NPP/VIIRS image scales in 2013 to DMSP/OLS image scales in 2013; the NOD in the y-axis represents normalized DN values of original DMSP/OLS image DN values in 2013.

**Fig. 2:**
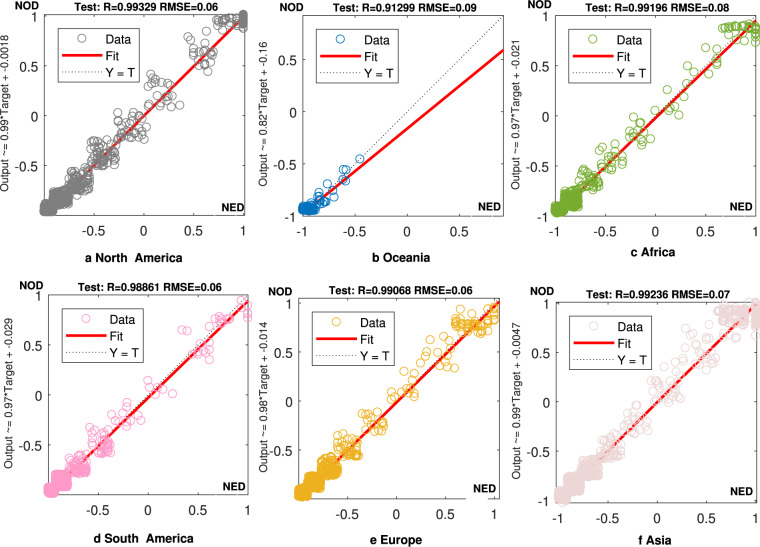
Different test results for the pixel normalized values on the six continents. (**a**–**f**). Test results for the pixel normalized values on (**a**) North America, (**b**) Oceania, (**c**) Africa, (**d**) South America, (**e**) Europe, and (**f**) Asia.

As shown in Fig. 1, all of the R^2^ of the six continents’ training results were > 0.96, indicating that the PSO-BP neural network performed well in identifying the potential relationship between DMSP/OLS and NPP/VIIRS images in 2013. The test results are shown in Fig. 2. The test performances of the six continents can be used to evaluate the prediction effects of the algorithm (i.e., whether the parameters of the PSO-BP algorithm can be employed to convert the pixel normalized DN values of NPP/VIIRS images during 2014–2019 to the scale of normalized DN values in DMSP/OLS images). Except for the fitting result of Oceania (i.e., the R^2^ was only 0.91), the fitting results of the other five continents all exceeded 0.98. The poor fitting effect of Oceania may be owing to the lack of light at night in most parts of Oceania. Considering Oceania has fewer stable light sources, its poor prediction results have limited impact on the matching of two sets of night lights at the global scale. Subsequently, the global converted normalized DN values of NPP/VIIRS images during 2013–2019 were denormalized to the original range, which was consistent with the scale of DN values in DMSP/OLS images. Moreover, the final global converted DN values of NPP/VIIRS images in 2013 and the DN values of DMSP/OLS images were compared again to verify the effect of matching: the global coefficient of determination was > 0.98, which was higher than that obtained in previous studies (for example, 0.91 in Zhao *et al*.^32^, 0.84 Lv *et al*.^33^, and 0.87 in Chen *et al*.^34^).

Based on the trained parameters of the neutral network, we transformed the scale of the NPP/VIIRS data from 2014 to 2019 to the scale of the DMSP/OLS data. As the generated network was based on “high to high” and “low to low” principle, the same pixels with high DN values in the NPP/VIIRS images can be converted into high DN values at the scale of DMSP/OLS images. However, the matching job was not complete yet. First, there were also certain pixels in NPP/VIIRS images with low DN values transformed into low DN values at DMSP/OLS scales, not matching the high DN values in the same regions of DMSP/OLS in 2013. Second, although the correlation coefficient was close to 1, there were evident and unavoidable discontinuity in some grids during 2013–2014, which also exist in previous studies^32^.

Therefore, inter-annual continuous series correction was adopted for the transformed NPP/VIIRS images from 2014 to 2019. In line with the correction approach, pixels with high DN values in the DMSP/OLS image were maintained in the converted NPP/VIIRS images for the period of 2014–2019. And the potential problem of discontinuity was solved. The equation is as follows:

1 $$\begin{array}{l}D{N}_{i,t}=\left\{\begin{array}{cc}D{N}_{i,t-1}, & if\,D{N}_{i,t-1}\ge D{N}_{i,t}\\ D{N}_{i,t}, & otherwise\end{array}\right.\left(t=2014,\; ..,2019\right),\\ \end{array}$$

**Fig. 3:**
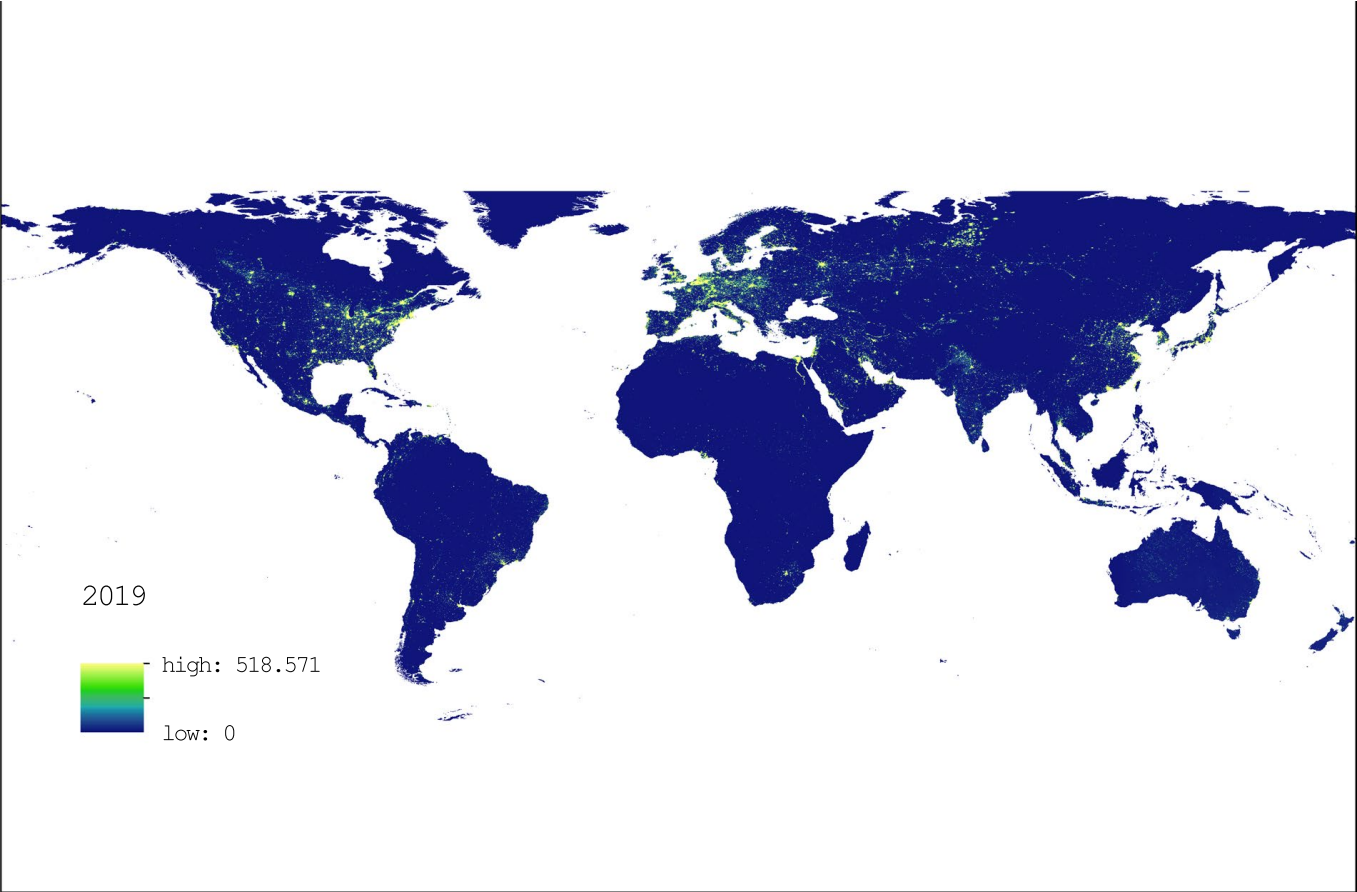
The 1 km × 1 km gridded nighttime light data in 2019.

In summary, based on the PSO-BP algorithm, we could confidently convert the scale of NPP/VIIRS data from 2013 to 2019 to the scales of DMSP/OLS data and obtain stable and continuous global 1 km × 1 km gridded nighttime light data for the time period of 1992–2019, which laid the foundation for further calculations of global 1 km × 1 km gridded GDP and electricity consumption during the period. Figure 3 presents the spatial distributions of global nighttime light data in 2019.

### Calculation of real GDP and electricity consumption based on growth rate

Owing to errors in official GDP growth attributed to poor statistical methods or intentional manipulation^10,11,21^, nighttime light data has been employed extensively in revision of official national GDP growth data. Based on the approaches proposed by Henderson *et al*.^10^ and Guerrero *et al*.^11^, the revised growth estimate is a composite with different weights of conventionally measured growth and growth predicted from nighttime light data. Considering the approaches of such studies, we employed nighttime light data to revise the real GDP growth rate. In particular, the real GDP growth rate was estimated using Eq. (2).

2 $${y}_{i,t}^{* }=\rho {y}_{i,t}+\left(1-\rho \right){y}_{i,t}^{{\prime} }$$

where ${y}_{i,t}^{* }$ is the *i*^*th*^ country’s real GDP growth in period *t*; ${y}_{i,t}$ is the official GDP growth of the *i*^*th*^ country in period *t*; ${y}_{i,t}^{{\prime} }$ presents the i^th^ country’s predicted GDP growth based on the night-time light data in period *t*; and $\left(1-\rho \right)$ is the optimal weight of predicted growth based on the night-time light data. In the light with the idea proposed by Henderson *et al*.^10^, the optimal value of *ρ* was specified to minimize the variance of measurement error in this estimate relative to the true value of GDP growth. As long as the optimal weight on $\left(1-\rho \right)$ is positive, use of night-time light data improves our ability to measure true GDP growth. The variance of this composite GDP growth was estimated by the following equation:

3 $$var\left({y}_{i}^{\wedge * }-{y}_{i}^{* }\right)={\rho }^{2}var\left({y}_{i}-{y}_{i}^{* }\right)+{\left(1-\rho \right)}^{2}\left({y}_{i}^{{\prime} }-{y}_{i}^{* }\right)$$

Following Henderson *et al*.^10^, the relationships between the night-time light data and real GDP growth/official GDP growth were described as the following equations:

4 $${y}_{i}={y}_{i}^{* }+{\varepsilon }_{y,i}$$

5 $$sdn{a}_{i}=\beta {y}_{i}^{* }+{\varepsilon }_{sdna,i}$$

6 $${y}_{i}=\gamma sdn{a}_{i}+{e}_{i}$$

7 $${\sigma }_{y}^{2}={\varepsilon }_{y,i}^{2}$$

8 $${\sigma }_{sdna}^{2}={\varepsilon }_{sdna,i}^{2}$$

where $sdn{a}_{i}$ is the growth of the sum of DN values per area; ${\varepsilon }_{y,i}$, ${\varepsilon }_{{sdna},i}$ and ${e}_{i}$ are the errors; *β* was is the elasticity of lights growth with respect to real GDP growth; *γ* was is the elasticity of official GDP growth with respect to lights growth; ${\sigma }_{y}^{2}$ and ${\sigma }_{sdna}^{2}$ are the variance of errors. Based on the assumption that the degree of measurement error in GDP growth has no effect on the estimated value of the parameter, there is $cov({\varepsilon }_{y},{\varepsilon }_{sdna})=0$. Thus, there were further derived equations as follows:

9 $$var(sdna)={\beta }^{2}{\sigma }_{y* }^{2}+{\sigma }_{sdna}^{2}$$

10 $$cov\left(sdna,y\right)=cov\left({y}^{* },sdna\right)=\beta {\sigma }_{y* }^{2}$$

11 $$var(y)={\sigma }_{y* }^{2}+{\sigma }_{y}^{2}$$

Then, the relationship between ${\gamma }^{\wedge }$ and the structural parameter *β* is as follows:

12 $$Plim\left({\gamma }^{\wedge }\right)=\frac{cov(sdna,y)}{var({\rm{sdna}})}=\frac{1}{\beta }\left(\frac{{\beta }^{2}{\sigma }_{y* }^{2}}{{\beta }^{2}{\sigma }_{y* }^{2}+{\sigma }_{sdna}^{2}}\right)$$

Thus, the Eq. (3) can be rewritten as follows:

13 $$var\left({y}_{i}^{\wedge * }-{y}_{i}^{* }\right)={\rho }^{2}{\sigma }_{y}^{2}+{\left(1-\rho \right)}^{2}\frac{{\sigma }_{sdna}^{2}{\sigma }_{y* }^{2}}{{\beta }^{2}{\sigma }_{y* }^{2}+{\sigma }_{sdna}^{2}}$$

From Eq. (13), we solve for the weight *ρ* which minimizes this variance:

14 $${\rho }^{* }=\frac{{\sigma }_{sdna}^{2}{\sigma }_{y* }^{2}}{{\sigma }_{y}^{2}\left({\beta }^{2}{\sigma }_{y* }^{2}+{\sigma }_{sdna}^{2}\right)+{\sigma }_{sdna}^{2}{\sigma }_{y* }^{2}}$$

Furthermore, following Henderson *et al*.^10^, *ρ* is further classified based on countries with good- and bad-quality data: ${\rho }_{i,good}$ and ${\rho }_{i,bad}$. Therefore, the Eq. (11) becomes two Eqs. (15, 16).

15 $$var({y}_{good})={\sigma }_{y* }^{2}+{\sigma }_{y,good}^{2}$$

16 $$var({y}_{bad})={\sigma }_{y* }^{2}+{\sigma }_{y,bad}^{2}$$

And the ratio of signal to total variance in official GDP growth for countries with good quality of statistics was estimated. A higher ratio of signal to total variance indicates more reliable GDP growth. The calculation equation was presented as follows:

17 $${\rm{\phi }}=\frac{{\sigma }_{{\rm{y}}* }^{2}}{{\sigma }_{{\rm{y}}* }^{2}+{\sigma }_{{\rm{y}},{\rm{good}}}^{2}},$$

where ϕ was set to 0.9 based on Henderson *et al*.^10^ and Guerrero *et al*.^11^. Therefore, ${\rho }_{i,good}$ and ${\rho }_{i,bad}$ can be determined with the following equations:

18 $${\rho }_{i,good}=\frac{{\sigma }_{sdna}^{2}{\sigma }_{y* }^{2}}{{\sigma }_{y,good}^{2}(\beta {\sigma }_{{y}^{* }}^{2}+{\sigma }_{SDNA}^{2})+{\sigma }_{SDNA}^{2}{\sigma }_{{y}^{* }}^{2}}$$

19 $${\rho }_{i,bad}=\frac{{\sigma }_{SDNA}^{2}{\sigma }_{{y}^{* }}^{2}}{{\sigma }_{y,bad}^{2}(\beta {\sigma }_{{y}^{* }}^{2}+{\sigma }_{SDNA}^{2})+{\sigma }_{SDNA}^{2}{\sigma }_{{y}^{* }}^{2}}$$

Considering the conclusions of previous studies^10,21,35^, statistics from developed countries always have better quality, while those from developing countries are less reliable. Therefore, we characterized the quality of a country’s data based on whether it is a developed country. In additions, the weights applied during growth prediction from nighttime light data (i.e., $\left(1-\rho \right)$) were different between developed and developing countries, which is consistent with Henderson *et al*.^10^. The classification into developed and developing countries was based on that of the United Nations (Statistics Division) provided by the World Bank^36^. Based on the above equations, we obtained the optimal weights of the official GDP growth rate in developed and developing countries (i.e., ${\rho }_{good}=0.94\;and\;{\rho }_{bad}=0.66$).

Furthermore, each grid’s real GDP growth rate during 1993–2019 can be estimated using the following equation:

20 $${{\rm{gy}}}_{ij,t}^{* }=\left\{\begin{array}{c}{\rho }_{gb}\times {y}_{i,t}+(1-{\rho }_{gb})\times \left(\frac{D{N}_{ij,t}-D{N}_{ij,t-1}}{D{N}_{ij,t-1}}\right)\times \alpha ,if\;D{N}_{ij,t-1}\ne 0\\ {y}_{i,t},if\;D{N}_{ij,t-1}=0\end{array}\right.,$$

where $g{y}_{ij,t}^{* }$ denotes the j^th^ grid in the i^th^ country’s real GDP growth; $gb=good,bad$; *α* represents the elasticity of the nighttime light data to GDP (i.e., 0.45 based on the regression results), which was obtained by Eq. (6).

Next, based on the gridded real GDP growth rate during 1993–2019, the gridded GDP data in 1992 or 2019 were estimated as basic values to obtain the gridded real GDP data in other years. Since the DN values in newly built-up areas were zero in 1992, these areas’ basic GDP values in 1992 were also zero, thereby leading to values of zero in subsequent years. Thus, the gridded GDP data in 2019 was selected as the basic value, which was calculated based on the top-down method.

Finally, the gridded real GDP based on the real growth rate can be calculated using Eq. (21).

21 $${{\rm{RGY}}}_{ij,t}^{* }=\left\{\begin{array}{c}\frac{{{\rm{RGY}}}_{ij,t+1}^{* }}{1+g{y}_{ij,t}^{* }},if\;D{N}_{ij,t}\ne 0\\ 0,if\;D{N}_{ij,t}=0\end{array},\right.$$

where $RG{Y}_{ij,t}^{* }$ denotes the *j*^*th*^ grid in the *i*^*th*^ country’s real GDP in the period of *t* based on the revised real growth rate. The calculations were based on the hypothesis that there is no GDP when the DN value is zero, which is consistent with Shi *et al*.^17^ and Wang *et al*.^16^.

As for electricity consumption, the gridded growth rate of nighttime light data was used to estimate the growth rate of gridded electricity consumption. However, because the growth rate of electricity consumption was mainly driven by the industrial sectors rather than the residential sector^37,38^, the growth rate of the nighttime light data may not comprehensively capture the growth rate of electricity consumption. Thus, we combined the growth of official GDP and nighttime light data to better reveal the gridded growth rate of electricity consumption, which is presented in Eq. (22).

22 $${\rm{ln}}E{C}_{it}=\gamma ln(SD{N}_{it})+\pi ln({Y}_{it})+{c}_{it}+{\tau }_{it},$$

where $E{C}_{it}$ denotes the *i*^*th*^ country’s electricity consumption in the period *t*, *SDN*_*it*_ denotes the *i*^*th*^ country’s sum of DN values in the period *t*, ${{\rm{c}}}_{it}$ denotes the constant, ${{\rm{\tau }}}_{it}$ denotes the errors, γ and π denote the coefficients (i.e., 0.22 and 0.71). Then, the gridded growth rate of the electricity consumption $GE{C}_{j,t}^{* }$ was calculated using Eq. (23).

23 $${{\rm{gecg}}}_{ij,t}^{* }=\left\{\begin{array}{c}\gamma \times \left(\frac{D{N}_{ij,t}}{D{N}_{ij,t-1}}-1\right)+\pi \times \left(\frac{{Y}_{i,t}}{{Y}_{i,t-1}}-1\right),if\;D{N}_{ij,t-1}\ne 0\\ 0,if\;D{N}_{ij,t-1}=0\end{array}\right.,$$

Given that only the worldwide electricity consumption during 1992–2015 was open-access and available freely, we selected the gridded electricity consumption data in 2015 as the basic values. Then, the gridded electricity consumption $GE{C}_{j,t}^{* }$ was calculated using Eq. (24).

24 $${{\rm{GEC}}}_{ij,t}^{* }=\left\{\begin{array}{c}\frac{GE{C}_{ij,t+1}}{1+{{\rm{gecg}}}_{ij,t}^{* }},if\;D{N}_{ij,t-1}\ne 0\\ 0,if\;D{N}_{ij,t-1}=0\end{array}\right.$$

With regard to the basic values of gridded GDP in 2019 and electricity consumption in 2015, we first established the relationships between national nighttime light data (i.e., the sum of the DN values) and targeted variables (i.e., GDP and electricity consumption) based on the top-down approach, respectively. Thus, the ratios of GDP and electricity to the nighttime light data (i.e., the coefficients of the targeted variables per unit of DN value) can be estimated among different countries (or regions) during 1992–2019, and each 1 km × 1 km grid can be assigned GDP and electricity consumption with the DN value as the weight. Thus, the ratios of GDP or electricity consumption to DN values were estimated using the following equations:

25 $${Y}_{it}^{* }={\beta }_{it}SD{N}_{it}+{\mu }_{it},$$

26 $$E{C}_{it}={\theta }_{it}SD{N}_{it}+{\epsilon }_{it},$$

where ${Y}_{it}^{* }$ represents the *i*^*th*^ country’s (or region’s) real GDP in the period *t*; ${\beta }_{it}$ and ${\theta }_{it}$ represent the coefficients of the *i*^*th*^ country’s (or region’s) in the period *t*; ${\mu }_{it}$ and ${\epsilon }_{it}$ denote the errors.

**Fig. 4:**
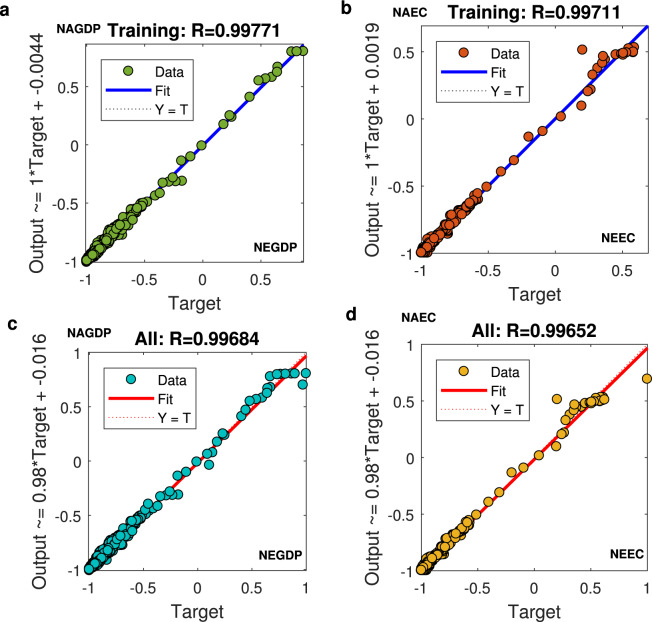
Training and all samples’ results for the relationship between national normalized actual GDP/ electricity consumption and our estimated GDP/electricity consumption predicted based on the input factors. (**a**–**d)**. (**a**) Training results for the relationship between national normalized actual GDP and our estimated GDP predicted based on the input factors; (**b**) Training results for the relationship between national normalized actual electricity consumption and our estimated electricity consumption predicted based on the input factors; (**c**) All samples’ results for the relationship between national normalized actual GDP and our estimated GDP consumption predicted based on the input factors; (**d**) All samples’ results for the relationship between national normalized actual electricity consumption and our estimated electricity consumption predicted based on the input factors.

Furthermore, in line with Chen *et al*.^31^, we employed the PSO-BP algorithm to fit and train the relationship among real GDP, electricity consumption, and nighttime light data. The real GDP and electricity consumption were selected as the output factors. The sum of DN values, dummy variables of identity and year were used as input parameters. In addition, the other initialized parameters were consistent with those discussed in the earlier section on the inter-calibration. According to the general practice in machine learning^28,31^, the input and output factors were normalized to avoid the influence of indicators’ units. The results are shown in Fig. 4. In particular, the NEGDP/NEEC in the x-axis represents our estimated national normalized GDP/electricity consumption predicted based on input factors; the NAGDP and NAEC in the y-axis represent national normalized actual GDP and electricity consumption, respectively.

Notably, the coefficients of determination R^2^ of normalized GDP and electricity consumption were over 0.99. Thus, the training and all samples’ results showed great fitting effects, which indicated the high effectiveness of the algorithm. Then, based on the top-down method and a DN value-based weighted-average strategy^39–41^, we obtained the 1 km × 1 km gridded GDP and electricity consumption in 2019 and 2015. Finally, the gridded real GDP and electricity based on the growth rate during 1992–2019 were calculated using Eqs. (21, 24).

## Data Records

**Fig. 5:**
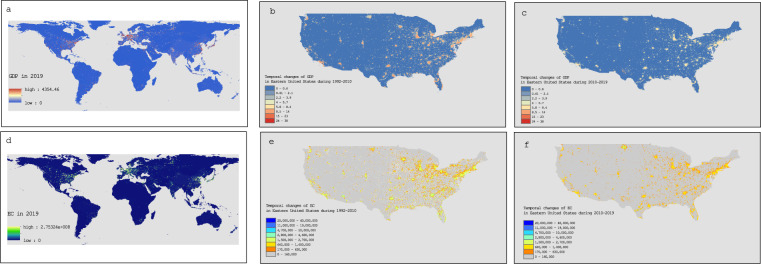
Global 1 km × 1 km gridded GDP and electricity consumption in 2019 and temporal changes of GDP and electricity consumption in Eastern United States during 1992–2010 and 2010–2019 (unit: millions of 2017 US dollar; kwh). (**a**–**f)**. (**a**) Global 1 km × 1 km gridded GDP in 2019; (**b**) temporal changes of GDP in Eastern United States during 1992–2010; (**c**) temporal changes of GDP in Eastern United States during 2010–2019; (**d**) Global 1 km × 1 km gridded electricity consumption in 2019; (**e**) temporal changes of electricity consumption in Eastern United States during 1992–2010; (**f**) temporal changes of electricity consumption in Eastern United States during 2010–2019.

A total of two sets of data records (gridded real GDP and electricity consumption) during 1992–2019 were calculated. The units for the estimated 1 km × 1 km gridded real GDP and electricity consumption data are millions of 2017 US dollars and kilowatt hours, respectively. The presented datasets and codes are publicly available under Figshare^42^,^43^. The global 1 km × 1 km gridded real GDP and electricity consumption emissions in 2019 are presented in Fig. 5(a),(d). To better present the detailed temporal changes of GDP and electricity during 1992–2019, we selected Eastern United States as samples, and Fig. 5(b,c,e,f) show the temporal changes of GDP and electricity consumption in Eastern United States during 1992–2019.

## Technical Validation

### Validity testing for the nighttime light data changes

**Fig. 6:**
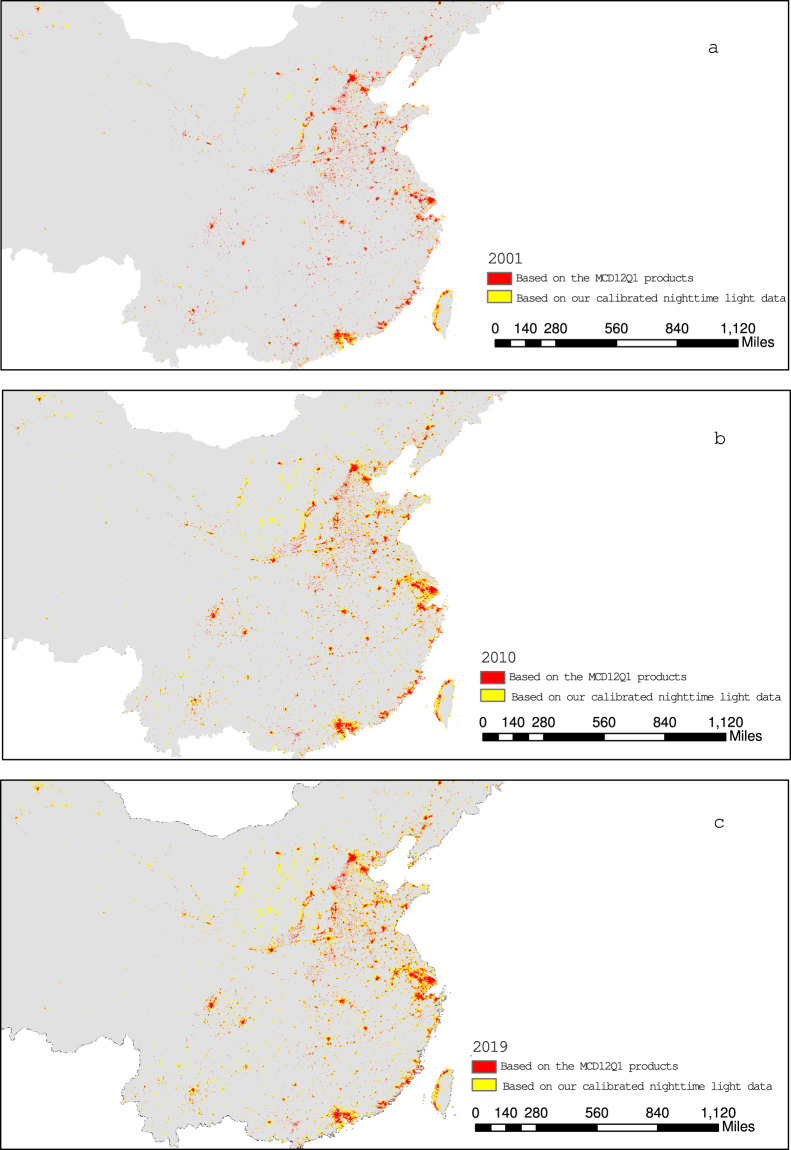
Comparison of urban built-up areas of China’s central and eastern regions derived from MCD12Q1 products and our calibrated nighttime light data in 2001, 2010 and 2019. (**a**–**c**). **(a**) The comparison of China’s central and eastern urban built-up areas derived from MCD12Q1 products and our calibrated nighttime light data in 2001. (**b**) The comparison of China’s central and eastern urban built-up areas derived from MCD12Q1 products and our calibrated nighttime light data in 2010. (**c**) The comparison of China’s central and eastern urban built-up areas derived from MCD12Q1 products and our calibrated nighttime light data in 2019.

Validity testing the spatial patterns of calibrated nighttime light data. Due to the strong relationship between the brightness of area of nighttime light and urbanization, nighttime light data has always been employed to extract built-up areas in urban development. Thus, we used the neighborhood boundary method proposed by Su *et al*.^40^ to extract the global urban built-up areas in 2001, 2010 and 2019 based on our calibrated nighttime light data. Next, the urban built-up areas provided by the MCD12Q1 products^44^ were selected as a reference to validate our calibrated nighttime light data in spatial patterns. Considering that the areas of urban built-up land in China’s central and eastern areas experienced quick increments, we selected their urban built-up areas as samples. Figure 6 presents the comparison of their urban built-up areas derived from the two sets of data.

It is evident that the urban built-up lands based on our calibrated nighttime light data were highly consistent with those derived from the MCD12Q1 products. It should be noted that the urban built-up area based on nighttime light data was relatively larger than that from MCD12Q1 products. Because urban built-up areas from the nighttime light data based on Su *et al*.^40^ considered the central urban region and the surrounding urban region, while the urban built-up land from MCD12Q1 products only identified approximately 30% of impervious surface areas (only including building materials, asphalt, and vehicles). The comparison results indicated that our calibrated nighttime light data performed well in the spatial patterns of urbanization.

Validity testing the spatial distributions of nighttime light data among the observed countries (or regions). Because nighttime light data tend to be highly consistent with economic output and electricity consumption, many scholars^45,46^ test the spatial distributions of region’s DN values by using the national cross-sectional GDP and electricity consumption to individually perform linear regressions with the sum of DN values (SDN). Following them, we also made regressions during 1992–2019. The results are shown in Table 1. It was evident that there was a significant positive relationship between national cross-sectional GDP and SDN during 1992–2019. The average R^2^ value was approximately 0.82 and 0.87. The results imply that our calibrated nighttime light data performed better in fitting the relationship between nighttime light data and GDP/electricity consumption. In addition, the AIC and BIC values were small, thereby implying that the inter-calibrated nighttime light data characterized the economic output well.

**Table 1:** Validity test results for the relationship between GDP/ electricity consumption and nighttime light data.

Year	GDP				Electricity consumption			
	Slope	R^2^	AIC value	BIC value	Slope	R^2^	AIC value	BIC value
1992	0.13***	0.82	28.71	28.75	3.75e + 07***	0.92	67.07	67.11
1993	0.12***	0.82	28.78	28.82	3.53e + 07***	0.91	67.15	67.20
1994	0.12***	0.81	28.87	28.91	3.40e + 07***	0.91	67.29	67.33
1995	0.12***	0.81	28.94	28.98	3.39e + 07***	0.91	67.32	67.37
1996	0.12***	0.81	29.02	29.06	3.39e + 07***	0.91	67.39	67.43
1997	0.12***	0.82	29.07	29.1	3.39e + 07***	0.91	67.43	67.47
1998	0.13***	0.82	29.09	29.13	3.39e + 07***	0.91	67.48	67.53
1999	0.13***	0.83	29.13	29.17	3.48e + 07***	0.91	67.52	67.56
2000	0.13***	0.84	29.19	29.23	3.55e + 07***	0.91	67.59	67.63
2001	0.14***	0.83	29.24	29.28	3.45e + 07***	0.90	67.61	67.66
2002	0.14***	0.84	29.28	29.32	3.53e + 07***	0.90	67.71	67.76
2003	0.14***	0.84	29.32	29.36	3.56e + 07***	0.89	67.85	67.89
2004	0.15***	0.85	29.38	29.42	3.63e + 07***	0.88	68.00	68.04
2005	0.15***	0.84	29.49	29.53	3.76e + 07***	0.87	68.71	68.75
2006	0.16***	0.84	29.59	29.63	3.81e + 07***	0.86	68.39	68.44
2007	0.16***	0.83	29.71	29.74	3.90e + 07***	0.83	68.64	68.69
2008	0.16***	0.83	29.74	29.77	3.91e + 07***	0.83	68.69	68.74
2009	0.16***	0.81	29.86	29.9	2.56e + 07***	0.91	67.10	67.15
2010	0.16***	0.81	29.97	30.01	3.68e + 07***	0.81	68.77	68.81
2011	0.16***	0.82	30.03	30.07	3.85e + 07***	0.80	68.98	69.03
2012	0.16***	0.82	30.07	30.11	3.91e + 07***	0.77	69.24	69.28
2013	0.17***	0.81	30.17	30.21	3.93e + 07***	0.76	69.33	69.37
2014	0.17***	0.8	30.33	30.36	4.05e + 07***	0.73	69.54	69.59
2015	0.17***	0.79	30.43	30.46	4.10e + 07***	0.72	69.65	69.94
2016	0.18***	0.79	30.53	30.57	—	—	—	—
2017	0.18***	0.78	30.61	30.64	—	—	—	—
2018	0.19***	0.79	30.63	30.67	—	—	—	—
2019	0.19***	0.78	30.71	30.74	—	—	—	—

Notes: *** significance at the 1% level. The slope represents the coefficients of the linear regression of the national cross-sectional GDP or electricity consumption with the sum of the DN values. The AIC denotes the Akaike information criterion. BIC represents the Bayesian information criterion.

### Validity Testing the PSO-BO algorithm for estimated GDP and electricity consumption

**Fig. 7:**
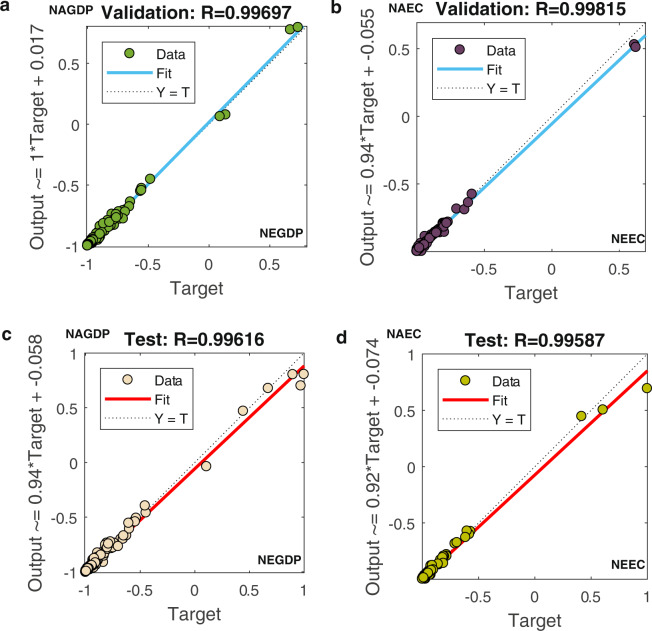
Validation and testing results for the relationship between national normalized actual GDP/ electricity consumption and our estimated GDP/ electricity consumption predicted based on the input factors. (**a**–**d)**. (**a**) Validation results for the relationship between national normalized actual GDP and our estimated GDP predicted based on the input factors; (**b**) Validation results for the relationship between national normalized actual electricity consumption and our estimated electricity consumption predicted based on the input factors; (**c**) Testing results for the relationship between national normalized actual GDP and our estimated GDP consumption predicted based on the input factors; (**d**) Testing results for the relationship between national normalized actual electricity consumption and our estimated electricity consumption predicted based on the input factors.

With regard to the validity of the PSO-BO algorithm for predicted GDP and electricity consumption based on nighttime light data, we first validate the effectiveness of the PSO-BO algorithm. Figure 7 shows the validation and testing results of the parameters in the PSO-BO algorithm. In particular, the NSDN in the x-axis represents national normalized sum of DN values; the NGDP and NEC in the y-axis represents national normalized GDP and electricity consumption, respectively. It is evident that all coefficients of determination R^2^ of GDP and electricity consumption are over 0.99, respectively. The results indicate that the employed PSO-BO algorithm was effective in our study.

**Fig. 8:**
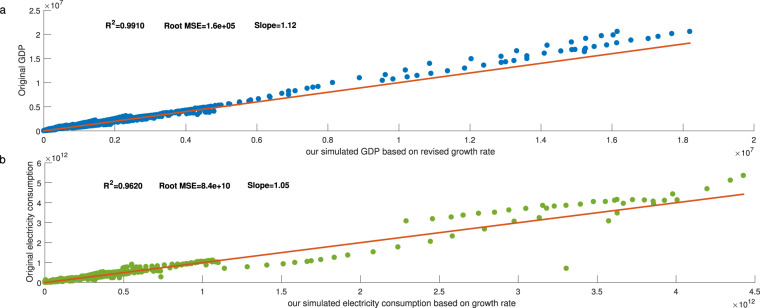
Scatter plots of our simulated national GDP and electricity consumption with the original data based on existing literature during 1992–2019. (**a**,**b)**. Scatter plots of our simulated (**a**) national GDP compared with the GDP and (**b**) national electricity consumption compared with the electricity consumption based on existing literature during 1992–2019.

Next, following Shi *et al*.^17^ and Chen *et al*.^31^, we utilized the original actual GDP^47^ and electricity consumption^17^ based on existing literature to conduct a comparison with the summary of our estimated GDP and electricity consumption. The results are shown in Fig. 8. Panels (a) and (b) in Fig. 8 individually show the scatter plots of our simulated national GDP and electricity with the data based on existing literature from 1992 to 2019. The results in each graph were highly consistent, indicating that there are no outliers in aggregated grid GDP and electricity consumption.

## Usage Notes

Since the 1 km × 1 km gridded GDP and electricity consumption were estimated based on the top-down idea, the scope of the gridded data did not involve all of the worldwide countries (175 countries’ or regions’ GDP data; 134 countries’ or regions’ electricity consumption). Thus, the values of the area beyond the scope of our study were set to 0. The official GDP data were derived from the Penn World Table, and the electricity consumption data were obtained from the World Bank. In addition, the projected coordinate system of all images was set as the Mollweide coordinate.

The provided datasets have the advantages of wide coverage and a long-time span. The datasets can help fill the existing data gaps and can be further used in future research. For example, gridded GDP data growth was revised based on nighttime light data, which is more objective and comparable, and more appropriate for use in research at a more micro-level in countries with poor quality statistics; at the same time, considering that our estimated electricity consumption data is more based on objective satellite data, it will be less affected by administrative intervention. Thus, the comparison between published electricity consumption and our estimated gridded electricity consumption based on satellite data can be employed to reveal failed/poorly governed states.

In addition, it should also be noted that our estimated gridded electricity consumption were based on the assumption that a more developed area generally has brighter lights and higher electricity consumption^15–17^. Thus, shocks like huge price fluctuations or other special events during a particular period were not considered in this study.

## Data Availability

The programs used to generate all the results were MATLAB (R2017b) and ArcGIS (10.5). The PSO-BP codes for modelling the relationships between the national GDP and nighttime light data are publicly available under Figshare^43^.
